# Patterns of adipose tissue fatty acids and the risk of atrial fibrillation: A case-cohort study

**DOI:** 10.1371/journal.pone.0208833

**Published:** 2018-12-11

**Authors:** Pia Thisted Dinesen, Thomas Andersen Rix, Albert Marni Joensen, Christina Cathrine Dahm, Søren Lundbye-Christensen, Erik Berg Schmidt, Kim Overvad

**Affiliations:** 1 Aalborg University Hospital, Department of Cardiology, Aalborg, Denmark; 2 Aalborg University Hospital, Aalborg AF Study Group, Department of Cardiology, Aalborg, Denmark; 3 Aarhus University, Department of Public Health, Section for Epidemiology, Aarhus, Denmark; 4 Aalborg University Hospital, Unit of Clinical Biostatistics, Aalborg, Denmark; 5 Aalborg University, Department of Clinical Medicine, Aalborg, Denmark; The Pennsylvania State University, UNITED STATES

## Abstract

Fatty acids in adipose tissue share dietary sources and metabolic pathways and therefore occur in patterns. The aim of the present study was to investigate the association between adipose tissue fatty acid patterns identified by the data-driven dimension-reducing method treelet transform and the risk of atrial fibrillation. A total of 57,053 Danish men and women aged 50–64 years participating in the Diet, Cancer and Health cohort had an adipose tissue biopsy taken at baseline. During a median follow-up of 14.6 years, a total of 4,710 participants developed atrial fibrillation or atrial flutter. Adipose tissue biopsies were analysed for fatty acid content by gas chromatography for all cases of atrial fibrillation and for a randomly drawn subcohort (n = 3,500) representative for the entire cohort. Hazard ratios with 95% confidence intervals for atrial fibrillation according to quintiles of factor scores were determined by weighted Cox proportional hazards regression analyses for men and women separately. From the 32 fatty acids measured, 7 major factors/patterns of fatty acids were identified using treelet transform. We found that a pattern consisting of n-6 polyunsaturated fatty acids (PUFA) (except linoleic acid) was associated with a lower hazard of atrial fibrillation. Patterns consisting of marine n-3 PUFA and containing n-9 fatty acids were associated with a lower hazard of atrial fibrillation in women. In conclusion, patterns of fatty acids in adipose tissue identified by treelet transform may be differentially associated with the risk of atrial fibrillation.

## Introduction

Atrial fibrillation (AF) is a common cardiac arrhythmia with a prevalence of 1–2% in the general population [1], which is believed to rise substantially due to the aging population, in addition to an increase in established risk factors for AF such as diabetes mellitus [2]. AF is associated with higher morbidity including a 5-fold higher risk of stroke and a 3-fold higher risk of congestive heart failure [1,3–5] and constitutes a serious public health problem.

Previous studies have suggested that certain fatty acids may be associated with development of AF. The mechanisms behind which fatty acids may effect development of AF is diverse and depends on the fatty acids involved. For saturated fatty acids the proposed mechanism has been apoptosis due to palmitic acid ceramide affecting permeability of the outer mitochondrial membrane [6–8], while marine n-3 polyunsaturated fatty acids (PUFA) have been suggested to have a rhythm-stabilising effect caused by effects on ion channels and membrane fluidity [9].

Previous studies have explored the potential associations for individual adipose tissue fatty acids but not the association between patterns of fatty acids in adipose tissue and development of AF [10,11]. As individual fatty acids share dietary sources and metabolic pathways, they are highly correlated. Fatty acid patterns in adipose tissue respect that individual fatty acids share dietary sources and metabolic pathways making the interpretation of data more clear than exploring individual fatty acids [12–15]. The statistical method treelet transform is a dimension-reduction method which takes correlations between variables into account and may be used to generate patterns of fatty acids contained in adipose tissue [16,17].

The purpose of this study was to investigate the association between fatty acid patterns in adipose tissue identified by the treelet transform and the long-term risk of AF in a Danish cohort.

## Material and methods

### Study population

The Danish Diet, Cancer and Health cohort was established between 1993 and 1997 and has previously been described in detail [18]. Briefly, 160,725 individuals born in Denmark, not registered with a diagnosis of cancer in the Danish Cancer Registry and aged 50–64 years received an invitation for participation, which was accepted by 57,053 subjects (29,875 women and 27,178 men).

**Fig 1:**
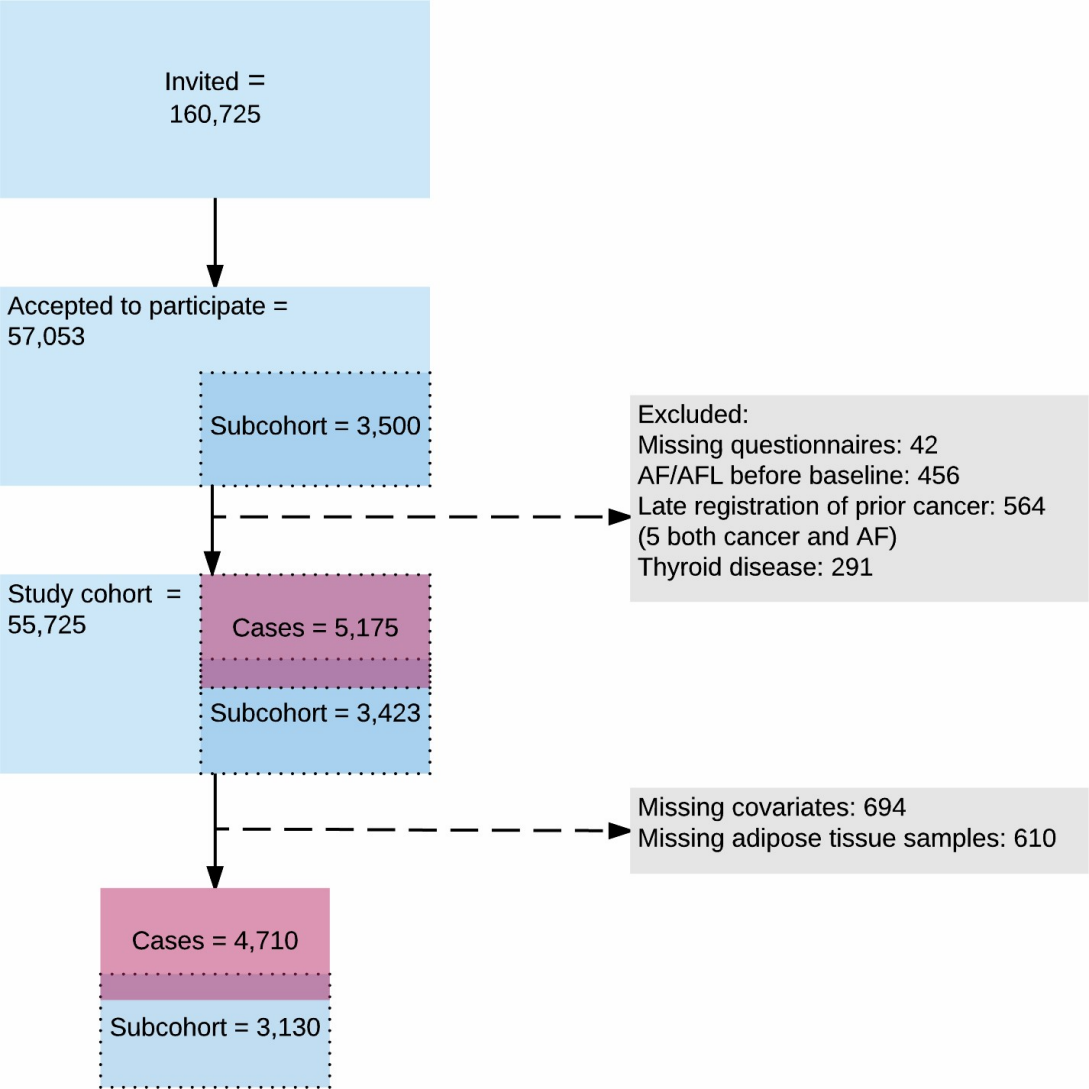
Flowchart of the participants in the Diet, Cancer and Health cohort study.

Participants answered questionnaires on diet, lifestyle, medications and health at baseline. Blood pressure and anthropometric measurements were performed, and biological material was collected. Participants with a delayed registration of cancer in the Danish Cancer Register were later excluded. For this study, participants with thyroid disease and/or documented AF/atrial flutter (AFL) prior to recruitment were also excluded (see Fig 1).

A randomly drawn subcohort of 3,500 participants representative for the entire cohort was used in this case-cohort study.

The study participants gave written informed consent and the research was conducted according to the Declaration of Helsinki. The Danish Data Protection Agency (project number: 2015–186) and the Ethics Committee in the North Denmark Region (project number: N-20160001) approved the study.

### Adipose tissue biopsy collection and analysis

At baseline, an adipose tissue biopsy was obtained from the buttocks according to the method described by Beynen and Katan [19]. As described previously [10], a luer lock system (Terumo, Terumo Corp, Tokyo, Japan) with a needle, a venoject multisample luer adaptor and an evacuated blood tube was used. The samples were flushed with nitrogen and stored at -150°C until analysis. When analysed, the biopsies were thawed and a small amount of adipose tissue was removed and heated at 50°C for 10 min. Subsequently, at 50°C, heptane dissolved the fat and the fatty acids were transesterified for 2 min with 2 mol/L KOH, according to the International Union of Pure and Applied Chemistry Standard Methods for the Analysis of Oils, Fats, and Derivatives. The fatty acid composition was determined by gas chromatography using a Varian 3900 GC with a CP-8400 autosampler (Varian, Middleburg, The Netherlands) equipped with a flame ionization detector. Constant flow, split injection mode and a CP-sil 88 60 m x 0.25-mm internal diameter capillary column and temperature programming from 90°C to 210°C were used. Helium was used as the carrier gas, and commercially available standards (Nu-Chek-Prep, Inc, Elysian, MN, US) were used to identify individual fatty acids [20]. Results for fatty acids were given as percentages of total fatty acids.

### Follow-up and outcome

Hospital discharge diagnoses have been registered in the Danish National Patient Register since 1977. By linking the personal identification number for all study participants with this register, information on AF/AFL development was obtained.

The 8^th^ International Classification of Diseases (ICD-8, code 427.4 in the international version, code 427.93/427.94 in the Danish version) was used until the end of 1993 and ICD-10 (code I.48.9x) subsequently. Follow-up was from baseline until first diagnosis of AF/AFL, emigration, death or until July 31^st^ 2013, whichever came first. The AF and AFL diagnoses have previously been validated in this cohort with a positive predictive value above 92% [21].

### Statistical analyses

In this case-cohort study, we used the treelet transform (TT) to derive sparse patterns of adipose tissue fatty acids [16]. TT is a data-driven dimension-reducing method that produce a number of factors/patterns, which account for much of the variation in the dataset. By creating factors across variables based on the correlation of fatty acids, fatty acid compositions can be expressed as patterns [16]. Not all fatty acids contribute to every pattern which eases the interpretation and separates TT from the more widely used method principal component analysis [17]. If an individual has a high score of a pattern this corresponds to that individual having an adipose tissue fatty acid profile that matches the factor loadings well.

The output of TT is a dendrogram/cluster tree where the branches contains related groups of variables. Patterns can be extracted by cutting the tree. Where to cut can be determined by cross-validation with a range of different factors.

TT was applied to the correlation matrix of 32 adipose tissue fatty acids in the subcohort. The optimal cut-off level was found according to a previously described method where a graph of cross-validation scores is generated, and a point at which increasing the cut-level does not substantially increase the cross-validation score, can be identified. Thus, the cut-level chosen is the level where we can explain as much variation as possible, while maintaining as low a cut-level as possible to simplify the interpretation of the patterns [22]. The factors were used to generate scores (magnitude of each pattern) for participants in the subcohort, as well as cases outside the subcohort according to each individual’s fatty acid profile.

Quintiles of the factor scores were used as exposure variables in weighted Cox proportional hazards regression analyses with age as the time axis and the possibility of delayed entry. Participants were treated as at risk from baseline until diagnosed with AF/AFL in the Danish National Patient Register, death, emigration or end of follow-up, whichever came first. Participants with missing adipose tissue biopsies or missing data in one or more covariates were excluded (Fig 1). In weighted Cox proportional hazards regression, used to account for the case-cohort design, weights were assigned to each participant [23]. Adipose tissue was analysed for all cases and thus, their assigned weight was one. For non-cases, adipose tissue for the randomly drawn subcohort was weighed as (non-cases in the cohort)/(non-cases in the subcohort). All models were weighted and analysed for men and women separately and adjusted for age at baseline in quintiles to ensure that the age of exposure data was comparable. We stratified by sex as sex-specific differences in pathophysiology and epidemiology for AF have been recognized [24]. Adjustments in the multivariate analyses were made for a list of predefined potential confounders registered at baseline including lifestyle factors such as smoking (never, former, <15, 15–25 or >25 g/day) and years in school (≤7 years, 8–10, ≥10 years) as categorical variables. Alcohol intake (g/day), body mass index (kg/m^2^) and waist circumference (cm) were adjusted for as continuous variables using restricted cubic splines with five knots (Model 1A). Model 2 was adjusted for the confounders in Model 1A and for comorbidities at baseline including history of hypercholesterolemia and/or cholesterol lowering treatment (yes/no), history of hypertension and/or treatment for hypertension (yes/no), angina pectoris (yes/no), heart failure (yes/no), previous myocardial infarction (yes/no), diabetes mellitus (yes/no) and renal disease (yes/no) as categorical variables. Data on diseases were retrieved from national registries.

The reported hazard ratio (HR) corresponds to a comparison between 2 subjects of the same sex with the same values of covariates.

Data were analysed using Stata statistical software (version 15, StataCorp LP, College Station, US) and the associated TT package [22].

## Results

A total of 4,710 participants developed documented AF during a median follow-up of 14.6 years. Baseline characteristics of the randomly drawn subcohort and all cases (including cases in the subcohort) are shown in Table 1. A greater number of men (n = 2,907) than women (n = 1,803) were cases. In the subcohort 271 participants developed AF (183 men and 88 women).

**Table 1:** Baseline characteristics of participants and cases.

	Men		Women	
	Subcohort (n = 1,693)	Cases (n = 2,907)	Subcohort (n = 1,437)	Cases (n = 1,803)
Age at entry (years)	56.3 (50.8;64.3)	58.1 (51.0;64.6)	56.3 (50.7;63.8)	59.3 (51.2;64.7)
Hypertension (self-reported)	15.3 (259)	22.6 (656)	16.8 (241)	26.6 (480)
Systolic blood pressure (mmHg)	140 (114;178)	144 (116;184)	136 (108;173)	142 (110:182)
Diastolic blood pressure (mmHg)	84 (69;103)	86 (70;105)	81 (66;98)	83 (67;100)
Body-mass index (kg/m^2^)	26.4 (22.2;33.0)	27.0 (22.2;35.2)	24.7 (19.8;34.2)	25.8 (20.2;36.8)
Waist circumference (cm)	96 (83;114)	98 (84;119)	80 (67;105)	83 (69;108)
Alcohol (g/day)	19.7 (1.4;79.5)	21.2 (1.8;84.7)	9.6 (0.5;41.4)	7.8 (0.2;44.2)
Hypercholesterolemia (self-reported)	9.5 (161)	9.6 (279)	5.9 (85)	8.9 (160)
Serum total cholesterol (mmol/L)	5.9 (4.4;7.8)	5.9 (4.3;7.9)	6.2 (4.5;8.4)	6.2 (4.5;8.5)
Smoking				
Never	26.8 (453)	25.8 (749)	44.8 (644)	41.0 (740)
Former	35.2 (595)	35.4 (1,030)	22.4 (322)	24.3 (438)
Current <15 g/day	11.2 (189)	10.2 (296)	15.9 (229)	15.5 (279)
Current 15–25 g/day	16.5 (279)	19.0 (552)	14.5 (209)	16.0 (289)
Current >25 g/day	10.5 (177)	9.6 (280)	2.3 (33)	3.2 (57)
Years in school >10 years	25.0 (423)	22.0 (638)	19.4 (279)	14.4 (259)
Angina pectoris (self-reported)	3.9 (66)	6.3 (182)	2.1 (30)	3.7 (67)
Diabetes mellitus	2.6 (44)	3.3 (97)	1.3 (19)	2.7 (48)
Renal disease	1.6 (27)	3.8 (110)	1.9 (27)	2.6 (47)
Previous myocardial infarction	3.0 (51)	4.2 (123)	0.3 (4)	1.2 (22)
Heart failure	0.2 (4)	0.6 (18)	0.1 (2)	0.5 (9)

% (number) or median (5th; 95th percentile).

**Fig 2:**
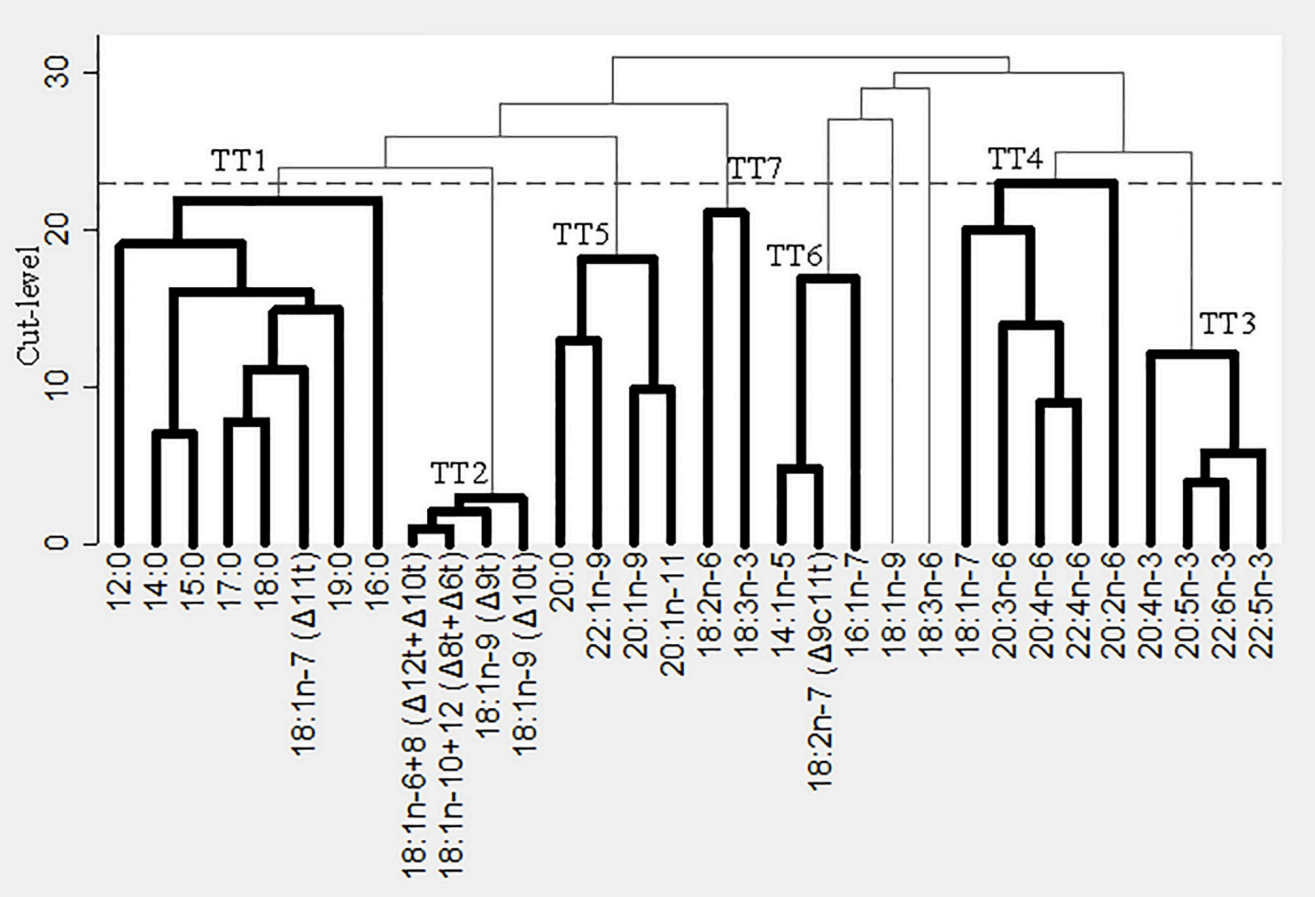
Treelet transform dendrogram of the 32 fatty acids in adipose tissue determined in the subcohort (n = 3,130). The horizontal line indicates the optimal cut at level 23.

TT analyses were performed and resulted in the treelet dendrogram/cluster tree shown in Fig 2. The optimal cut-level for the cluster tree was found to be 23 and the optimal number of factors to retain was seven. The 7 patterns cumulatively explained 66% of the fatty acid variance in the subcohort and contained 8 fatty acids at most. Of the 32 fatty acids in the analysis all, except C18:1 n-9 and C18:3 n-6, were contained in one pattern (Table 2). TT1 contained saturated fatty acids (SFA), TT2 consisted of trans fatty acids, TT3 consisted of marine n-3 PUFA, TT4 contained n-6 PUFA (except linoleic acid), TT5 contained n-9 fatty acids, TT6 consisted of minor products of stearoyl-CoA desaturase and TT7 consisted of the plant-based n-3 PUFA α-linolenic acid and the n-6 PUFA linoleic acid. TT factor scores were divided into quintiles for men and women and hazard ratios (HR) were calculated for each factor with the first quintile as the reference (Table 3 (men) and Table 4 (women)).

**Table 2:** Fatty acid loadings on treelet transform factors.

Fatty acid	Common name	Mean	SD	Factor loading						
				TT1	TT2	TT3	TT4	TT5	TT6	TT7
12:0	Lauric acid	0.4	0.1	0.3						
14:0	Myristic acid	2.7	0.5	0.4						
14:1n-5	Nonadecanoic acid	0.4	0.1						0.6	
15:0	Pentadecanoic acid	0.3	0.1	0.4						
16:0	Palmitic acid	20.2	2.0	0.2						
16:1n-7	Palmitoleic acid	6.6	1.6						0.5	
17:0	Heptadecanoic acid	0.2	<0.1	0.4						
18:0	Stearic acid	3.5	1.0	0.4						
18:1n-6+8 (Δ12t+Δ10t)*		0.3	0.1		0.5					
18:1n-9 (Δ9t)	Elaidic acid	0.6	0.2		0.5					
18:1n-8 (Δ10t)		0.3	0.1		0.5					
18:1n-7 (Δ11t)	Vaccenic acid	0.3	0.1	0.4						
18:1n-10+12 (Δ8t+Δ6t)*		1.5	0.4		0.5					
18:1n-9	Oleic acid	44.0	2.0							
18:1n-7	Asclepic acid	2.0	0.4				0.4			
18:2n-7 (Δ9c11t)	Rumenic acid	0.4	0.1						0.6	
18:2n-6	Linoleic acid	10.7	1.9							0.7
18:3n-3	α-linolenic acid	0.8	0.2							0.7
18:3n-6	γ-linolenic acid	0.1	<0.1							
19:0	Nonadecanoic acid	0.1	<0.1	0.4						
20:0	Arachidic acid	0.2	0.1					0.5		
20:1n-9	n-9 eicosenoic acid	0.8	0.2					0.5		
20:1n-11	n-11 eicosenoic acid	0.2	0.1					0.5		
20:2n-6	Eicosadienoic acid	0.2	<0.1				0.3			
20:3n-6	Dihomo-γ-linolenic acid	0.2	0.1				0.5			
20:4n-3	Eicosatetraenoic acid	0.1	<0.1			0.5				
20:4n-6	Arachidonic acid	0.4	0.1				0.5			
20:5n-3	Eicosapentaenoic acid	0.1	<0.1			0.5				
22:1n-9	Eurcic acid	0.1	<0.1					0.5		
22:4n-6	Docosatetraenoic acid	0.1	<0.1				0.5			
22:5n-3	Docosapentaenoic acid	0.3	0.1			0.5				
22:6n-3	Docosahexaenoic acid	0.3	0.1			0.5				
Explained variance (%)				14.2	11.7	10.0	9.2	8.7	7.4	4.6

Standard deviation, SD

Mean percentage of fatty acids in adipose tissue (% of total fatty acids) and standard deviation, their loadings on treelet transform factors and the explained variance of extracted factors for participants in the subcohort (n = 3,130).

*The peaks for 18:1n-6 (Δ12t) and 18:1n-8 (Δ10t) and 18:1n-10 (Δ8t) and 18:1n-12 (Δ6t) could not be separated in the analysis

**Table 3:** Results for men.

Fatty acid pattern	Factor score quintile	HR (95% CI)		
		Model 1*	Model 1A^†^	Model 2^‡^
TT1	1.	1.00 (ref.)	1.00 (ref.)	1.00 (ref.)
*Saturated fatty acids*	2.	0.80 (0.65–0.97)	0.92 (0.75–1.12)	0.99 (0.81–1.22)
	3.	0.88 (0.73–1.07)	1.06 (0.87–1.30)	1.14 (0.93–1.40)
	4.	0.67 (0.56–0.82)	0.89 (0.72–1.09)	0.97 (0.78–1.19)
	5.	0.57 (0.47–0.70)	0.82 (0.65–1.02)	0.89 (0.71–1.11)
	P(trend)	0.0000	0.11	0.41
TT2	1.	1.00 (ref.)	1.00 (ref.)	1.00 (ref.)
*Trans fatty acids*	2.	0.91 (0.75–1.11)	1.00 (0.82–1.24)	1.02 (0.83–1.25)
	3.	0.88 (0.72–1.07)	1.04 (0.84–1.29)	1.07 (0.86–1.33)
	4.	0.95 (0.78–1.16)	1.10 (0.89–1.36)	1.14 (0.91–1.40)
	5.	0.94 (0.77–1.14)	1.20 (0.96–1.52)	1.25 (0.99–1.58)
	P(trend)	0.71	0.08	0.03
TT3	1.	1.00 (ref.)	1.00 (ref.)	1.00 (ref.)
*Marine n-3 PUFA*	2.	1.40 (1.15–1.71)	1.25 (1.01–1.53)	1.29 (1.05–1.60)
	3.	1.18 (0.97–1.44)	1.02 (0.83–1.26)	1.05 (0.85–1.30)
	4.	1.35 (1.11–1.65)	1.22 (0.99–1.50)	1.23 (0.99–1.53)
	5.	1.07 (0.87–1.31)	0.93 (0.75–1.16)	0.95 (0.76–1.19)
	P(trend)	0.83	0.37	0.40
TT4	1.	1.00 (ref.)	1.00 (ref.)	1.00 (ref.)
*n-6 PUFA*	2.	0.72 (0.60–0.87)	0.66 (0.54–0.81)	0.65 (0.53–0.80)
	3.	0.72 (0.60–0.88)	0.60 (0.49–0.73)	0.60 (0.49–0.74)
	4.	0.85 (0.71–1.03)	0.62 (0.50–0.77)	0.58 (0.46–0.72)
	5.	0.98 (0.81–1.18)	0.54 (0.43–0.68)	0.48 (0.38–0.61)
	P(trend)	0.85	0.0000	0.0000
TT5	1.	1.00 (ref.)	1.00 (ref.)	1.00 (ref.)
*n-9 fatty acids*	2.	0.95 (0.78–1.16)	1.05 (0.86–1.30)	1.12 (0.90–1.39)
	3.	0.91 (0.75–1.11)	1.09 (0.88–1.35)	1.15 (0.92–1.43)
	4.	0.93 (0.76–1.12)	1.16 (0.94–1.44)	1.24 (0.99–1.55)
	5.	0.71 (0.58–0.87)	0.92 (0.72–1.17)	0.99 (0.77–1.25)
	P(trend)	0.0027	0.92	0.67
TT6	1.	1.00 (ref.)	1.00 (ref.)	1.00 (ref.)
*Stearoyl-CoA desaturase*	2.	0.98 (0.80–1.19)	0.96 (0.78–1.18)	0.95 (0.77–1.18)
	3.	1.12 (0.92–1.37)	1.12 (0.92–1.38)	1.16 (0.95–1.43)
	4.	1.12 (0.92–1.36)	1.06 (0.86–1.30)	1.04 (0.85–1.29)
	5.	1.24 (1.02–1.52)	1.12 (0.91–1.38)	1.17 (0.95–1.44)
	P(trend)	0.01	0.16	0.09
TT7	1.	1.00 (ref.)	1.00 (ref.)	1.00 (ref.)
*α-linolenic acid*	2.	0.76 (0.63–0.93)	0.84 (0.68–1.04)	0.81 (0.66–1.00)
*Linoleic acid*	3.	0.74 (0.61–0.90)	0.85 (0.68–1.05)	0.84 (0.68–1.04)
	4.	0.82 (0.67–1.00)	0.99 (0.80–1.22)	0.97 (0.78–1.20)
	5.	0.95 (0.78–1.15)	1.19 (0.96–1.47)	1.18 (0.96–1.47)
	P(trend)	0.91	0.03	0.03

Confidence interval, CI

Polyunsaturated fatty acids, PUFA

Hazard ratio (HR) with 95% confidence interval for developing atrial fibrillation in quintiles with the first quintile as the reference. P-value for trend.

*Adjusted for age at baseline in quintiles

^†^Adjusted for model 1 and body mass index (kg/m^2^), waist circumference (cm), smoking (never, former, <15, 15–25, >25 g/day), alcohol (g/day), years in school (≤7 years, 8–10 years, ≥10 years)

^‡^Adjusted for model 1A and for hypertension (yes or medication, no, not known), hypercholesterolemia (yes or medication, no, not known), diabetes mellitus (yes, no), angina pectoris, previous myocardial infarction (yes, no), heart failure (yes, no), renal disease (yes, no)

**Table 4:** Results for women.

Fatty acid pattern	Factor score quintile	HR (95% CI)		
		Model 1*	Model 1A^†^	Model 2^‡^
TT1	1.	1.00 (ref.)	1.00 (ref.)	1.00 (ref.)
*Saturated fatty acids*	2.	0.72 (0.57–0.90)	0.78 (0.61–1.00)	0.80 (0.63–1.03)
	3.	0.84 (0.67–1.06)	0.99 (0.77–1.26)	1.03 (0.80–1.32)
	4.	0.73 (0.58–0.93)	0.92 (0.71–1.18)	0.92 (0.71–1.19)
	5.	0.57 (0.45–0.73)	0.76 (0.58–0.99)	0.80 (0.61–1.05)
	P(trend)	0.0001	0.22	0.34
TT2	1.	1.00 (ref.)	1.00 (ref.)	1.00 (ref.)
*Trans fatty acids*	2.	1.07 (0.84–1.36)	1.13 (0.88–1.46)	1.15 (0.88–1.50)
	3.	1.31 (1.03–1.66)	1.47 (1.14–1.91)	1.59 (1.21–2.07)
	4.	1.08 (0.85–1.37)	1.27 (0.98–1.66)	1.39 (1.06–1.82)
	5.	1.07 (0.84–1.36)	1.30 (0.99–1.72)	1.44 (1.08–1.91)
	P(trend)	0.65	0.06	0.01
TT3	1.	1.00 (ref.)	1.00 (ref.)	1.00 (ref.)
*Marine n-3 PUFA*	2.	0.86 (0.67–1.10)	0.82 (0.64–1.06)	0.82 (0.64–1.06)
	3.	1.07 (0.85–1.36)	0.94 (0.74–1.21)	0.96 (0.75–1.23)
	4.	0.98 (0.77–1.24)	0.83 (0.64–1.07)	0.82 (0.63–1.07)
	5.	0.71 (0.56–0.91)	0.63 (0.48–0.82)	0.60 (0.45–0.78)
	P(trend)	0.04	0.002	0.0007
TT4	1.	1.00 (ref.)	1.00 (ref.)	1.00 (ref.)
*n-6 PUFA*	2.	1.05 (0.83–1.34)	0.93 (0.72–1.19)	0.91 (0.70–1.17)
	3.	1.08 (0.85–1.38)	0.86 (0.66–1.11)	0.82 (0.63–1.06)
	4.	1.07 (0.85–1.36)	0.77 (0.59–1.00)	0.73 (0.56–0.96)
	5.	0.98 (0.77–1.25)	0.55 (0.41–0.75)	0.47 (0.35–0.64)
	P(trend)	0.92	0.0001	0.0000
TT5	1.	1.00 (ref.)	1.00 (ref.)	1.00 (ref.)
*n-9 fatty acids*	2.	0.68 (0.54–0.86)	0.76 (0.59–0.98)	0.77 (0.60–1.00)
	3.	0.66 (0.53–0.84)	0.77 (0.60–1.00)	0.80 (0.61–1.04)
	4.	0.58 (0.46–0.74)	0.68 (0.51–0.89)	0.73 (0.55–0.96)
	5.	0.55 (0.44–0.70)	0.70 (0.52–0.93)	0.72 (0.54–0.97)
	P(trend)	0.0027	0.01	0.03
TT6	1.	1.00 (ref.)	1.00 (ref.)	1.00 (ref.)
*Stearoyl-CoA desaturase*	2.	1.00 (0.78–1.27)	1.05 (0.82–1.35)	1.07 (0.83–1.39)
	3.	1.00 (0.79–1.27)	1.05 (0.82–1.35)	1.10 (0.85–1.42)
	4.	1.09 (0.86–1.38)	1.19 (0.92–1.52)	1.22 (0.95–1.57)
	5.	1.07 (0.84–1.35)	1.16 (0.91–1.50)	1.23 (0.95–1.59)
	P(trend)	0.41	0.13	0.07
TT7	1.	1.00 (ref.)	1.00 (ref.)	1.00 (ref.)
*α-linolenic acid*	2.	0.87 (0.68–1.10)	0.87 (0.68–1.12)	0.88 0.68–1.12)
*Linoleic acid*	3.	0.90 (0.71–1.14)	0.91 (0.71–1.16)	0.90 (0.70–1.15)
	4.	0.85 (0.67–1.08)	0.84 (0.65–1.09)	0.84 (0.65–1.09)
	5.	0.99 (0.78–1.25)	1.00 (0.78–1.29)	1.00 (0.78–1.29)
	P(trend)	0.91	0.95	0.94

Confidence interval, CI

Polyunsaturated fatty acids, PUFA

Hazard ratio (HR) with 95% confidence interval for developing atrial fibrillation in quintiles with the first quintile as the reference. P-value for trend.

*Adjusted for age at baseline in quintiles

^†^Adjusted for model 1 and body mass index (kg/m^2^), waist circumference (cm), smoking (never, former, <15, 15–25, >25 g/day), alcohol (g/day), years in school (≤7 years, 8–10 years, ≥10 years)

^‡^Adjusted for model 1A and for hypertension (yes or medication, no, not known), hypercholesterolemia (yes or medication, no, not known), diabetes mellitus (yes, no), angina pectoris, previous myocardial infarction (yes, no), heart failure (yes, no), renal disease (yes, no)

We consider Model 1A the primary model as this was adjusted for lifestyle factors. For men (Table 3) there were a neutral association between SFA (TT1) and development of AF. The trans fatty acids pattern (TT2) did not reach a statistically significant association in any of the quintiles and the p-value for trend was also non-significant (0.08). The marine n-3 PUFA pattern (TT3) was not associated with the hazard of AF. The n-6 PUFA pattern (TT4) was associated with a statistically significant lower hazard of AF in quintiles 2–5 compared to the first quintile, which was supported by a p-value of 0.0000 for trend. For the n-9 fatty acid pattern (TT5) and stearoyl-CoA desaturase products (TT6) we found no consistent associations. For the α-linolenic acid and linoleic acid (TT7) pattern there may be an association between α-linolenic acid and linoleic acid and a higher hazard of AF with a p-value for trend of 0.03, while none of the quintiles reached statistical significance.

Among women (Table 4), no clear association for saturated fatty acids was found. There were no statistically significant association for trans fatty acids and AF when considering the individual hazard ratios but the p-value for trend was 0.06. For the marine n-3 PUFA pattern a negative association was observed. However, when exploring the quintiles, only the HR in quintile 5 was statistically significant associated with development of AF. For the n-6 PUFA pattern, the p-value for trend was 0.0001 and the HR for quintile 5 compared to the first quintile was 0.55 (95% CI 0.45 to 0.75) suggesting a lower hazard of AF, while none of the other quintiles reached statistical significance. In the n-9 fatty acids pattern the HR for quintile 5 compared to the first quintile was 0.70 (95% CI 0.52 to 0.93), also quintile 2 and 4 was associated with a lower risk of AF compared to quintile 1, and the p-value for trend was 0.01 suggesting a lower hazard of AF. For TT6 and TT7 neutral associations with AF was observed.

## Discussion

This large study of 4,710 cases of AF investigated patterns of fatty acids in adipose tissue and the future risk of developing AF.

Our study had several strengths: We studied the naturally occurring adipose tissue fatty acid patterns and the risk of incident AF in a large cohort with long and almost complete follow-up for vital status. Also, the follow-up used national registries and the diagnoses of AF in the cohort were validated with a positive predictive value >92% [21]. Instances of misclassification of AF cases were unlikely to be associated with the exposure and would therefore lead to an underestimation of associations between fatty acid patterns and the risk of AF. Due to the large number of cases in both men and women, we were able to study the association with incident AF in both sexes separately.

The study also had some limitations: The study participants were 50–64 years old at enrolment and therefore the results may not be valid in other age groups. Also, participants were all Caucasians so the results may not be valid for other ethnicities.

As in other cohort studies the highest degree of participation was among participants with higher education. Also, married people were more likely to participate than single or cohabiting persons. However, due to the present study being a case-cohort design non-participants would only cause bias if the non-response was related to both the exposure, here adipose tissue content of fatty acids, and also to the risk of the outcome of the study, which seems highly unlikely [18].

We did not have information regarding the use of specific medications such as statins or fibrates among participants in the cohort during follow-up. However, to our knowledge there is no indication that these drugs affect the fatty acid composition in adipose tissue.

Adipose tissue samples were only obtained once. Thus, a change in the participants’ dietary habits and metabolism could potentially change the composition of fatty acids in adipose tissue, which this study was unable to capture. However, it is plausible that these middle-aged participants had a fairly stable diet. Also, we used subcutaneous adipose tissue from the buttocks. Previous studies have suggested that the content of fatty acids may vary according to the location of the subcutaneous adipose tissue. E.g. adipose tissue from the buttock may contain more MUFA and less SFA than subcutaneous abdominal adipose tissue [13,14,25]. Furthermore, for example epicardial adipose tissue might have different metabolic properties than subcutaneous adipose tissue concerning development of AF [26].

A recently published study [27] found higher levels of n-3 PUFA in the myocardium of diabetic patients compared to age-matched controls free from diabetes mellitus. In our study, we adjusted the results for diabetes mellitus but did not have sufficient statistical power to analyse data based on diabetes mellitus status at baseline.

We considered including physical activity but data on physical activity in this cohort were sparse and previous studies on the cohort have shown that adjustment for physical activity does not affect the results substantially. Finally, there is always a possibility of residual confounding.

We used TT to derive patterns of fatty acids in adipose tissue as TT has previously been used in the medical literature to create patterns of dietary data [17,28] and patterns of adipose tissue content of fatty acids [29–31].

Several other methods have been suggested and applied for pattern analysis: principal component analysis, factor analysis [32] and, more recently, structural equation modelling [33].

Each method has distinct strengths, limitations and assumptions. All these methods are unsupervised, meaning the constructed patterns solely reflect the distribution in the population in question without taking into account the potential association with study endpoint. Thus, there is no guarantee of an associations between factors and the endpoint, although some of the single variables contributing to a factor may be strong risk factors.

When constructing the cluster tree (dendrogram) the main assumption is the distinct correlation structure with high correlation within factors and low correlations between factors.

TT identifies factors based on the sample correlation. These correlations may be attenuated by the presence of analytical noise. This can be managed in methods with latent variables such as factor analysis and structural equation modelling. However, the TT cluster tree is produced based on the order of correlations and not their actual magnitude. If the analytical noise is reasonably independent and homogeneous, the order of correlations would be similar to absence of analytical noise.

The sparsity of TT factors is simple to interpret, hence we have chosen this method as the explorative technique used in this study. In the future, further confirmatory analyses could be of interest.

The grouping of fatty acids in patterns may reflect shared dietary sources and metabolism within the body, where the fatty acids compete for enzymes responsible for desaturation and elongation of fatty acids [34]. In this study TT7 reflects shared dietary sources. A previous study using principal component analysis on seven fatty acids in adipose tissue found that the factors were based on the chemical structure of the fatty acids [35], which was also the case in the present study. To our knowledge, no previous studies have evaluated the association between adipose tissue patterns of fatty acids and the risk of AF. By using 32 adipose tissue fatty acids and deriving patterns using TT, our study was able to condense information on these adipose tissue fatty acids and determine the most correlated patterns prevalent in this cohort study, while retaining a simple, sparse, structure to the extracted factors. Many of the fatty acids in the extracted factors have not been described in relation to AF previously, and our study contributes to expanding the literature for trans fatty acids, n-6 PUFA and n-9 fatty acids, in particular. Clinicians and basic researchers can use this study, when considering whether these fatty acids are related to the risk of AF. Furthermore, as fatty acids occur in patterns, it is not possible to investigate the association between individual fatty acids and the risk of diseases independent of the pattern the fatty acid occur in.

The existing literature regarding fatty acid patterns and the risk of developing AF, will be discussed below in relation with the results of the present study.

Regarding SFA (TT1), previous studies have suggested that SFA replacing carbohydrates in the diet and higher circulating plasma levels of C16:0 were associated with a higher risk of AF [36,37]. In this study, we could not confirm an association between an adipose tissue pattern high in SFA and a higher hazard of AF. Also, we have previously found in this cohort that replacing dietary SFA with n-3 PUFA was associated with a moderately higher risk of AF in men, while replacement with MUFA or other PUFA did not affect the risk [38]. Recently, we also explored the association between adipose tissue content of the major individual SFA (myristic (C14:0), palmitic (16:0) and stearic (C18:0) acid) and the risk of AF and did not find an association [10]. In contrast, other investigators have reported that higher circulating plasma levels of C16:0 were associated with a higher risk of AF in the highest compared to the lowest quartile, while circulating levels of the longer chained SFA C18:0-C24:0 were associated with a lower risk of AF [36]. However, as the present study explored patterns based on statistical correlations and the other studies explored individual SFA or hypothesis-driven SFA groups (based on common dietary sources and length of the carbon chain) the results cannot be directly compared.

Regarding an adipose tissue pattern with marine n-3 PUFA (TT3) a previous study on the subcohort (179 cases of AF) used in our study showed that marine n-3 fatty acids in adipose tissue were monotonic, negatively associated with development of AF, but the association was not statistically significant [11]. Why an association was only found in women in this study is unknown, but it might be explained by differences in metabolic processes caused by the different sex hormones, albeit most women in the cohort were postmenopausal at enrolment [24].

As previously mentioned, we found no previous studies on trans fatty acids (TT2), n-6 PUFA (TT4) or n-9 fatty acids (TT5), and the risk of developing AF. The minor products of stearoyl-CoA desaturase (TT6) were not significantly associated with the hazard of AF. The major product of stearoyl-CoA desaturase is oleic acid (C18:1 n-9) which is the most abundant fatty acid in adipose tissue and not included in any factors [13,34]. TT derives factors balancing interpersonal variation and variable correlation. Thus, abundant fatty acids such as oleic acid may not vary greatly between individuals and therefore will not be retained in a factor. However, the minor products in TT6 may show greater variation between individuals and will therefore be retained. The dietary intake of palmitoleic acid (C16:1 n-7) is low but is relatively abundant in adipose tissue due to endogenous synthesis [39] and our neutral findings suggest that palmitoleic acid is not important for the development of AF. A pattern with α-linolenic and linoleic acid (TT7) showed a trend towards a higher hazard of AF in men after adjustment for lifestyle, but not in women. Previous studies have not found an association with either dietary intake, or plasma or serum concentrations of α-linolenic acid and the risk of AF [40,41]. Furthermore, in the Framingham Heart Study, a combined dietary intake of arachidonic and linoleic acid was not associated with AF [42].

## Conclusions

We here report associations between patterns of fatty acids in adipose tissue identified by TT and the risk of developing AF. In both men and women, patterns with n-6 PUFA (TT4) may be associated with a lower hazard of AF. Patterns with marine n-3 PUFA (TT3) and n-9 fatty acids (TT5) were associated with a lower hazard of AF in women, while in men, patterns with α-linolenic acid and linoleic acid (TT7) may be associated with a higher hazard of AF.
